# EEG Oscillatory Dynamics During Real-World Goal-Oriented Praxis Actions (GOPAs)

**DOI:** 10.3390/brainsci16050441

**Published:** 2026-04-22

**Authors:** Michela Balconi, Benedetta Vignati, Flavia Ciminaghi, Laura Angioletti

**Affiliations:** 1 International research center for Cognitive Applied Neuroscience (IrcCAN), Università Cattolica del Sacro Cuore, 20123 Milan, Italybenedetta.vignati1@unicatt.it (B.V.);; 2 Research Unit in Affective and Social Neuroscience, Department of Psychology, Università Cattolica del Sacro Cuore, 20123 Milan, Italy

**Keywords:** goal-oriented praxis action, EEG, executive functions, mental workload

## Abstract

**Highlights:**

- The measurement of EEG oscillatory activity during ecological goal-directed praxis actions reveals dynamic modulation of goal-directed actions by complexity and hierarchical task structure, with more complex conditions and tasks eliciting stronger neural responses.
- Frontal theta power tracks executive demands, whereas posterior beta–gamma activity supports sensorimotor integration in complex praxis actions.

**Abstract:**

**Background/Objectives**: Goal-directed praxis actions (GOPAs) integrate perception, motor planning, and executive control. While neural correlates of single actions are known, less is understood about how complexity conditions and their hierarchical organization into elementary tasks shape neural dynamics during ecologically manual assembly tasks. This study tested how electrophysiological (EEG) activity reflects global complexity and selective engagement of executive and sensorimotor systems across GOPAs. **Methods**: 38 healthy young adults completed two assembly conditions differing in complexity (basic and advanced) decomposed into four elementary tasks: identification, handling, alignment, and joining. EEG was recorded across five frequency bands (delta, theta, alpha, beta, and gamma) and four regions of interest (ROI): frontal, fronto-central, temporo-central, and parieto-occipital. **Results**: Neural activity varied significantly depending on different complexity, elementary task, and ROI. The advanced-complexity condition elicited stronger neural responses compared to the basic-complexity condition, reflecting greater cognitive, and sensorimotor demands. A task-related gradient emerged, with joining showing the highest activity, followed by alignment, while identification and handling showed lower activation. Frontal regions, particularly in theta activity, were more involved under higher complexity, suggesting increased executive control. In contrast, beta and gamma activity predominated in temporo-central and parieto-occipital regions, supporting visuomotor and sensorimotor integration. **Conclusions**: EEG oscillatory dynamics during ecological GOPAs are selectively modulated by complexity condition and hierarchical task organization. Neural activity tracks functional demands of specific action phases rather than general arousal, highlighting dynamic coordination between executive and sensorimotor systems during complex manual behavior.

## 1. Introduction

Goal-directed praxis actions (GOPAs) constitute a fundamental component of human behavior, enabling individuals to interact effectively with objects and tools to produce specific effects in the environment. Within cognitive neuroscience, such actions are conceptualized as intentional behaviors guided by internal goals rather than simple stimulus–response associations [1,2]. Their execution requires the integration of perceptual-cognitive processing, motor planning, and executive control processes, supported by fronto-parietal networks involved in action representation, prediction, and implementation [3]. These networks allow the translation of perceptual information into appropriate higher-order motor commands while continuously monitoring action outcomes (e.g., gestures) [4]. Crucially, GOPA relies on executive functions (EFs), including working memory, inhibitory control, cognitive flexibility, and performance monitoring. These processes enable the maintenance of overarching goals, the selection of context-appropriate motor schemas, the inhibition of irrelevant action, tendencies, and the flexible updating of action plans in response to environmental feedback. Thus, praxis behavior does not merely involve motor execution but depends on the dynamic coordination of executive and sensorimotor mechanisms [5,6].

A defining characteristic of goal-directed action is its hierarchical organization. Complex actions are not executed as unitary motor programs but are structured as ordered sequences of elementary sub-actions, each contributing to the achievement of an overarching goal [7]. This hierarchical structure enables flexible adaptation to changing task demands and environmental constraints, allowing motor components to be reorganized while preserving overarching action goals across different complexity conditions [8]. Such hierarchical control inherently recruits EFs, as the coordination of elementary sub-actions requires goal maintenance, sequencing, monitoring, and the regulation of cognitive resources. This continuous interdependence between cognition and action can be observed in everyday praxis behaviors, making it essential to clarify which executive mechanisms are engaged across different stages of manual action execution.

For investigating the neural mechanisms underlying hierarchical GOPAs, an ecologically valid framework is provided by the object manipulation and manual assembly conditions. These actions require the sequential coordination of object identification, manipulation, spatial alignment, and component integration, thereby engaging both cognitive and motor processes across multiple execution stages [4,9]. Importantly, task demands and action complexity modulate the recruitment of neural resources, highlighting the dynamic interaction between executive control and sensorimotor integration during extended action sequences [3]. Assembly complexity is defined here as the combined physical and mental effort required to successfully complete the action.

While physical workload has been extensively studied, the ex-ante assessment of mental workload and executive demands in manual assembly and praxis actions remains relatively unexplored [10]. Certain action phases, such as planning, spatial alignment, or integration of object components, are likely to impose greater executive demands, particularly in terms of working memory updating, attentional control, and monitoring, whereas other phases may rely more heavily on automated sensorimotor processes [11]. Consequently, neural modulations associated with increased mental workload are expected to emerge selectively rather than globally, reflecting the dynamic allocation of executive and motor resources across different stages of action. Despite extensive research on isolated action components, previous studies have rarely examined how neural dynamics unfold across multiple, sequential stages of goal-directed manual assembly, particularly under varying complexity conditions [12,13]. As a result, it remains unclear how executive and sensorimotor processes are selectively recruited during each elementary sub-action in ecologically valid tasks. The present study addresses this gap by systematically comparing two assembly tasks differing in complexity (basic vs. advanced) and by decomposing each task into four functionally distinct elementary actions: identification, alignment, joining, and handling [14]. This approach allows investigation of whether EEG oscillatory activity reflects the hierarchical organization of goal-directed actions and the selective engagement of cognitive and motor systems. By integrating hierarchical models of action [1,8] with neuroergonomic frameworks of cognitive workload [15,16], the study provides a concrete rationale for examining electroencephalography (EEG) dynamics across task stages, rather than treating manual assembly as a unitary process, and highlights the novelty of linking task complexity, action decomposition, and distributed cortical activity in a real-world assembly context.

Importantly, this selective recruitment of neural resources aligns with a neuroergonomic view of praxis, in which goal-directed actions are supported by distributed neural networks that dynamically integrate executive control, sensorimotor processing, and perceptual representations of objects and tools, rather than by uniform changes in overall neural activation [15]. Likewise, goal-oriented actions are best understood as emerging from coordinated activity within fronto-parietal systems rather than isolated cortical regions, enabling flexible adaptation to task demands, environmental constraints, and tool-related challenges commonly encountered in real-world contexts [16].

Consistent evidence from cognitive neuroscience and neuroergonomics indicates a functional differentiation between anterior and posterior cortical regions during goal-directed manual actions. Anterior areas, particularly within the prefrontal and premotor cortices, are primarily involved in action planning, performance monitoring, and executive regulation, including the allocation of attentional resources and the management of mental workload [15,17]. Conversely, posterior regions, encompassing parietal and occipito-temporal cortices, play a central role in sensorimotor integration, spatial processing, and object-related representations that support accurate manipulation and tool use [3,8].

Among neuroscientific methodologies, EEG offers high temporal resolution and is widely employed to investigate the neural dynamics underlying action execution in both controlled laboratory and applied contexts. Within this framework, EEG frequency bands are not interpreted as direct indicators of discrete cognitive functions; rather, their modulation reflects context-sensitive variations in cognitive control, attentional engagement, and sensorimotor processing. Low-frequency oscillations, especially theta and alpha rhythms, have been associated with sustained attention, executive control, and mental effort [18,19,20]. Within hierarchical action models, increasing structural complexity entails greater demands on goal maintenance, sequencing, monitoring, and coordination of subordinate motor schemas. Frontal theta oscillations have consistently been linked to such executive and monitoring processes [1,8,15,16,18,21,22].

Whereas higher-frequency activity in the beta and gamma ranges has been linked to motor planning, sensorimotor coordination, and the fine-tuning of goal-directed movements [22,23]. In particular, beta and gamma activity in temporo-central and posterior regions has been associated with sensorimotor integration and action implementation [3,16].

Regardless of the substantial body of literature addressing the neural organization of action, prior research has largely examined isolated components of this phenomenon, focusing on single actions, specific frequency bands, or restricted cortical regions. Although such studies have provided robust and replicable findings, they offer a partial account of the intrinsically multilevel and dynamic nature of praxis behavior, particularly when considered in relation to EFs [24]. Comprehensive approaches that simultaneously integrate complexity conditions, constituent elementary tasks [13,14], EEG spectral dynamics, and the spatial distribution of cortical activity within ecologically valid tool-mediated scenarios remain relatively scarce.

Based on this reference literature, the present study examined the neurophysiological mechanisms underlying the execution of two ecological GOPAs differing in complexity condition (i.e., basic and advanced). The two assembly GOPAs’ conditions varied in the number of required action steps, the quantity and configuration of components, and the associated mental workload. Each GOPA was decomposed into functionally distinct elementary tasks: identification (I; object recognition and selection), handling (H; manipulation and stabilization), alignment (A; spatial positioning and adjustment), and joining (J; integration of components into a coherent structure) [13]. By integrating hierarchical models of goal-directed action [1,8] with neuroergonomic frameworks of cognitive workload [15,16], the current study aimed to determine whether EEG oscillatory brain dynamics reflect (i) global increases in complexity and (ii) selective recruitment of executive and sensorimotor systems depending on complexity condition and tasks’ role in GOPAs. To do so, EEG activity was examined across multiple frequency bands and predefined cortical Regions of Interest (ROIs) to capture the distributed neural architecture of action control. On these experimental bases, the following hypotheses were formulated.

First, we hypothesized that the advanced-complexity compared to the basic-complexity manual assembly condition would be associated with increased frontal theta power, reflecting enhanced cognitive control and monitoring demands [15,23], together with enhanced beta and gamma activity in temporo-central and parieto-occipital regions, indexing greater sensorimotor integration during manual assembly [3,22].

The decomposition of GOPAs into elementary tasks (i.e., I, A, H, and J) further allowed testing whether EEG oscillatory dynamics mirror the functional organization of the action sequence. Given their differentiated cognitive and motor requirements, they were hypothesized to exhibit distinct frequency- and region-specific profiles. In particular, J, which requires integration, coordination, and online monitoring, was conceptualized as engaging executive and sensorimotor systems more extensively than the other tasks involving more circumscribed operations. Secondly, we predicted that the complexity condition would selectively amplify neural activity during the most cognitively demanding tasks, rather than uniformly affecting all of them [11].

Finally, oscillatory modulation was examined in relation to the anterior–posterior functional organization of cortical control. Hierarchical rostro-caudal models posit that anterior regions support abstract goal representation and cognitive control, whereas posterior regions mediate sensorimotor transformation and execution [3,14]. Consistent with this framework, lower-frequency oscillations, particularly theta band, were hypothesized to show predominant frontal modulation, while higher-frequency bands (beta and gamma) were expected to be more strongly observed in temporo-central and posterior regions during assembly behavior.

These hypotheses were tested within a unified statistical framework, in which complexity, task, and ROI factors were modeled to directly map onto global EEG neural effects, hierarchical task-related modulation, and spatial-functional differentiation, respectively.

## 2. Materials and Methods

### 2.1. Sample

The sample consisted of 38 healthy young adults (Mage = 20.53, SD = 1.3; 18 females) and was recruited through convenience sampling within an academic environment. To determine the minimum required sample size for a repeated-measure MANOVA within-subject design, an a priori power analysis was conducted using G* Power 3.1. Assuming a medium effect size (f^2^(V) = 0.25), an alpha level of 0.05, and a desired power of 0.80, the required minimum sample size was approximately 36 participants. The final sample of 38 participants was therefore adequate to detect the expected effects. The exclusion criteria included traumatic head injury and/or ictus, clinically significant distress and depression, neurologic or psychiatric illness, and ongoing therapy with psychoactive medications affecting cognition and decision-making, as assessed through a semi-structured interview.

Participants showed low familiarity with assembly tasks (mean = 1.78, SD = 0.52, on a five-point Likert scale) and were predominantly right-handed, as assessed by the Edinburgh Handedness Inventory [25].

All participants were over 18 years of age, healthy, and had normal or corrected-to-normal vision and hearing, as well as no history of severe shoulder, elbow, wrist, hand, or lower back pain and an absence of motor or musculoskeletal disorders that could impair manual performance. All experimental procedures complied with the Declaration of Helsinki (2013) and with the General Data Protection Regulation (GDPR; EU Reg. 2016/679). Ethical approval was obtained from the Ethics Committee of Politecnico di Torino (CER-Polito). Written informed consent was obtained from all participants prior to the experiment.

### 2.2. Experiment Procedure

The experiment was conducted in a quiet, light-controlled laboratory environment. Upon arrival, participants received a detailed explanation of the study procedures and completed a demographic questionnaire. Continuous EEG activity was recorded throughout the experimental session, including a 2 min resting-state baseline (eyes open) followed by assembly execution. Participants performed two GOPAs with different levels of complexity, designed to investigate the neurocognitive processes underlying goal-directed manual action. Participants were instructed to complete the assembly task as accurately as possible, ensuring that each component was correctly positioned and assembled according to the required procedure. Emphasis was placed on minimizing errors and maintaining a consistent level of performance throughout the task. The GOPAs required participants to assemble physical objects and were chosen because they require coordinated engagement of perceptual, motor, and executive functions, while at the same time representing a highly familiar and ecologically valid form of goal-directed action in both everyday and work-related environments.

**Figure 1:**
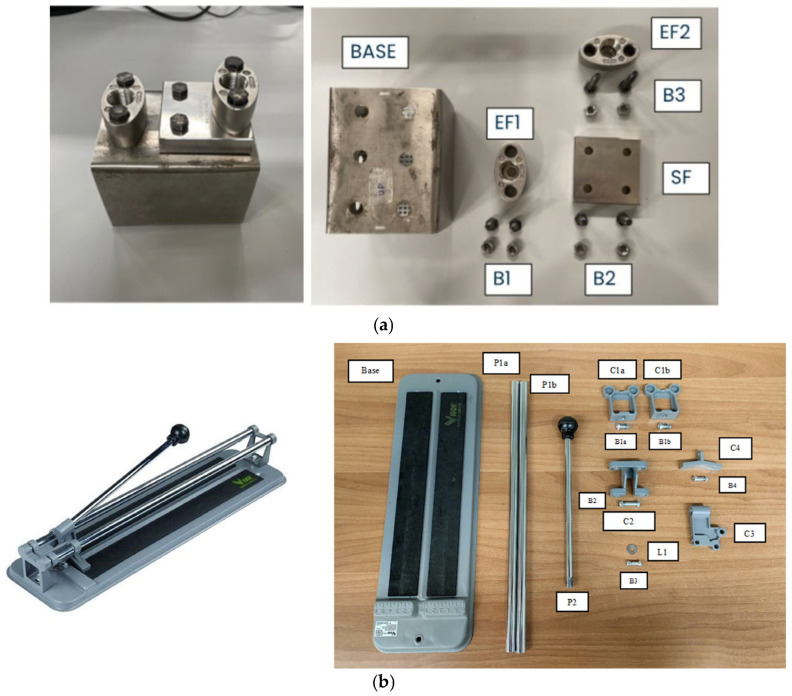
(**a**) Mechanical components assembled and their components corresponding to the basic complexity condition. (**b**) Tile cutter assembled and its components corresponding to the advanced complexity condition.

Established complexity metrics, such as the number of parts, step variability, and degree of decision-making, have been used to manipulate the complexity of the two GOPAs, with the more complex one demanding greater cognitive effort and engagement of EFs [13,14]. Two GOPAs were therefore administered: (i) a basic-complexity condition, involving the assembly of a mechanical equipment (Figure 1a), characterized by a limited number of components, fewer sequential steps, and minimal decision-making requirements and (ii) a advanced-complexity condition, involving the assembly of a tile cutter (Figure 1b), which required a larger number of components, more action repetitions, and increased demands for monitoring, sequencing, and online decision-making [10].

Both GOPAs were completed by each participant in a randomized order. Prior to the experimental trials, participants completed two training trials to familiarize themselves with task demands and object properties. Due to the intrinsic difference in complexity, the average completion time differed between conditions, with the basic assembly lasting approximately 2 min and the advanced assembly lasting approximately 5 min.

In addition, to allow neurocognitive characterization of the GOPAs, the assembly process was further decomposed into four functional elementary tasks, each reflecting a distinct action goal and set of cognitive demands [13,14], with the first task corresponding to the lowest hierarchical level and the last task corresponding to the highest hierarchical level:

- Identification (I): the visual recognition and selection of the appropriate component, discrimination between similar elements (e.g., screws of different lengths).
- Handling (H): planning and execution of grasping and transport actions, including movement trajectory planning and object manipulation.
- Alignment (A): detection of the correct spatial orientation of one or more components and their precise alignment.
- Joining (J): physical connection of components through the application of appropriate force and motor control.

These elementary tasks occurred repeatedly and in variable order during the assembly process, with their frequency and duration naturally varying as a function of complexity and individual performance.

After the conditions, a set of post-assembling questionnaires was filled out to assess the subjective experience perceived by participants. Specifically, the NASA-TLX [26] was used to assess the perceived mental workload (total workload score).

The mechanical component assembly consists of the following components:

- BASE—This is the perforated base of the assembly, and there are two pieces included.
- EF1—The first oval flange, which comes as a single piece.
- EF2—The second oval flange, also supplied as one piece.
- SF—The square flange, included as one piece.
- V1—Short hex head screws, four pieces in total, used for fastening EF1 and SF.
- V2—Long hex head screws, two pieces in total, used for fastening EF2.
- D—Hex nuts, six pieces included, used together with the screws.
- B1—Bolts consisting of V1 screws and nuts, used to join EF1 to the base, with a quantity of two.
- B2—Bolts consisting of V1 screws and nuts, used to join the square flange (SF) to the base, with a quantity of two.
- B3—Bolts consisting of V2 screws and nuts, used to join EF2 to the square flange, with a quantity of two.

The assembly process of the mechanical component can be structured around four sequential phases corresponding to the elementary tasks (I, H, A, and J). These phases integrate all components involved, from the base to the second oval flange, in a continuous workflow.

1. Identification:

During this phase, the operator recognizes and selects all necessary components for the assembly. This includes the perforated base (BASE), the first and second oval flanges (EF1, EF2), the square flange (SF), short and long screws (V1, V2), and the corresponding nuts (N). Proper identification ensures that each component is correctly prepared for the subsequent handling and assembly operations.

2. Handling:

Once identified, the components are manipulated and positioned for assembly. The base (BASE) is placed on the workbench near the operator, while the flanges (EF1, SF, EF2) are lifted and positioned roughly in their designated locations. Screws and nuts are also handled, brought to the assembly area, and prepared for insertion. This phase ensures that all parts are accessible, safely positioned, and ready for precise alignment.

3. Alignment:

In the alignment phase, each component is precisely oriented relative to the others. The first oval flange (EF1) is aligned with the base holes, the square flange (SF) is aligned on the base, and the second oval flange (EF2) is aligned on top of the square flange. Screws and nuts are similarly positioned in the correct holes, ensuring that all mating surfaces and fasteners are properly oriented for secure joining.

4. Joining:

Finally, all components are mechanically fastened together. Short screws (V1) with nuts (N) are used to secure EF1 and SF to the base, while long screws (V2) with nuts (N) attach EF2 to the square flange. This phase completes the assembly, ensuring structural integrity and stability. Upon completion, all connections are verified to guarantee that the assembly meets design specifications.

The tile cutter assembly consists of the following components:

- Base—Tile cutter base plate—Quantity: 1;
- C1—Side support for guide rods—Quantity: 2;
- V1—Screw for fastening C1a and C1b—Quantity: 2;
- N1—Nut for fastening C1a and C1b—Quantity: 2;
- B1—Bolt (V1 + N1) for joining C1—Quantity: 2;
- C2—Connector component between guide rods and cutting mechanism—Quantity: 1;
- N2—Screw for fastening C3—Quantity: 1;
- D2—Self-locking nut for fastening C3—Quantity: 1;
- B2—Bolt (V2 + D2) for joining C2 with C3—Quantity: 1;
- C3—Component of the cutting mechanism—Quantity: 2;
- L1—Washer blade—Quantity: 2;
- V3—Screw for fastening L1 and C4—Quantity: 2;
- R1—Metal ring—Quantity: 2;
- N3—Self-locking nut for fastening L1 and C4—Quantity: 2;
- B3—Bolt (V3 + R1 + D3) for joining L1—Quantity: 2;
- C4—Cutting mechanism component for breaking the tile—Quantity: 1;
- B4—Bolt (V3 + R1 + D3) for joining C4—Quantity: 1;
- P1—Guide rod of the tile cutter—Quantity: 2;
- P2—Handle of the tile cutter—Quantity: 1.

The assembly of the tile cutter can be structured around four sequential phases corresponding to the elementary tasks: I, H, A, and J. These phases integrate all components of the product, from the base to the handle, in a continuous workflow.

1. Identification:

In this phase, the operator identifies all necessary components for the assembly. This includes the tile cutter base plate (Base), side supports (C1), connector component (C2), cutting mechanism components (C3 and C4), washer blades (L1), metal rings (R1), screws and nuts (V1, V2, V3, N1, N2, and N3), bolts (B1, B2, B3, and B4), guide rods (P1), and handle (P2). Accurate identification ensures that each component is correctly prepared for subsequent manipulation and positioning.

2. Handling:

Once identified, the components are moved and positioned for assembly. The base plate is placed in the assembly area near the operator. The side supports (C1) are lifted and positioned on the base. Screws and nuts are brought to the assembly area and prepared for insertion. Cutting mechanism components (C2, C3, and C4) and blades (L1) are handled and positioned for joining. Finally, the guide rods (P1) and handle (P2) are placed in proximity to the assembly. This phase ensures that all parts are accessible and ready for precise alignment.

3. Alignment:

During this phase, components are precisely oriented relative to each other. The side supports (C1) are aligned with the base, the cutting mechanism components (C2, C3, C4) are aligned to fit together, and the washer blades (L1) and metal rings (R1) are correctly positioned within the mechanism. Guide rods (P1) are aligned with their housings in the cutting mechanism and side supports. The handle (P2) is aligned with the threaded hole in C3. Proper alignment ensures accurate assembly and reliable mechanical connections.

4. Joining:

In the final phase, all components are mechanically fastened to complete the assembly. Screws (V1, V2, and V3) with their respective nuts (N1, N2, and N3) and bolts (B1, B2, B3, and B4) are tightened to secure the side supports, cutting mechanism components, and blades. The guide rods (P1) are locked into place, joining the cutting mechanism to the base. Finally, the handle (P2) is fastened, completing the tile cutter assembly. Upon completion, all connections are verified to ensure structural integrity and operational functionality.

### 2.3. EEG Data Acquisition and Preprocessing

EEG signals were continuously recorded using a 32-channel portable EEG (LiveAmp from Brain Products, GmbH, Gliching, Germany) during both the resting-state baseline and assembly execution. An ElectroCap outfitted with silver/silver chloride (Ag/AgCl) electrodes was placed on the scalp according to the international 10/20 positioning system [27]. The electrode setup comprised 32 channels: Fp1, Fp2, F7, F3, Fz, F4, F8, FT9, FC5, FC1, FC2, FC6, FT10, C3, Cz, C4, T7, T8, TP9, CP5, CP1, CP2, CP6, TP10, P7, P3, Pz, P4, P8, O1, Oz. Fpz served as ground and FCz as reference. Signals were sampled at 500 Hz, with a 50 Hz notch filter applied online to suppress electrical line noise. Electrode impedances were kept below 5 kΩ.

Using a bandpass filter of 0.01–250 Hz, a sampling rate of 500 Hz, and a 50 Hz notch input filter, data were gathered using the BrainVision Recorder program (Brain Products GmbH, Munich, Germany). Impedance was controlled and kept below 5 kΩ. To gather information about ocular movements, an EOG electrode was placed on the eye canthi. Regarding EEG signal analysis, a 0.5–45 Hz IIR filter with a slope of 48 dB/octave was used to filter offline resting-state and task-related data. Based on synchronized video recordings, EEG segments corresponding to each occurrence of the four elementary tasks (I, H, A, J) were manually identified. After segmenting the data, ocular examination was used to check for any remaining ocular, muscular, or movement artifacts. Segments contaminated by eye movements, muscle artifacts, or excessive noise were excluded following visual inspection. Criteria for rejection included: (i) peak-to-peak amplitude > ±80 µV, (ii) abrupt non-physiological transients, (iii) eye-blink or eye-movement contamination affecting frontal electrodes, or (iv) sustained high-frequency activity indicative of muscle artifacts. Across participants, the proportion of rejected data was low, with comparable rates across conditions (I = 5%, H = 5%, A = 2%, J = 5%). Bad channels were identified based on abnormal signal characteristics (e.g., excessive noise or flat signals) and were interpolated using spherical spline interpolation. Subsequently, the data were re-referenced to the common average reference (CAR). Only epochs free from artifacts were taken into account to enhance signal specificity. For each segment, power spectral density (PSD) was computed using Fast Fourier Transform (FFT) with a Hamming window and 0.5 Hz frequency resolution. To accommodate variable segment durations, segments shorter than 2 s were excluded, and longer segments were divided into consecutive 2 s epochs. PSD was computed for each epoch and then averaged across all epochs within a segment. Five canonical frequency bands were analyzed: Delta (0.5–3.5 Hz), Theta (4–7.5 Hz), Alpha (8–12.5 Hz), Beta (13–30 Hz), and Gamma (30.5–50 Hz). For each participant and electrode, PSD values were normalized relative to the eyes-open resting-state baseline according to PSD_norm = (PSD_task − PSD_baseline)/PSD_baseline. Baseline PSD was computed using 2 min eyes-open recordings, which underwent identical artifact rejection, filtering, and FFT processing as task segments to ensure methodological consistency and comparability across conditions. For each participant, normalized PSD values were averaged across all occurrences of a given elementary task, producing one mean PSD value per task.

**Figure 2:**
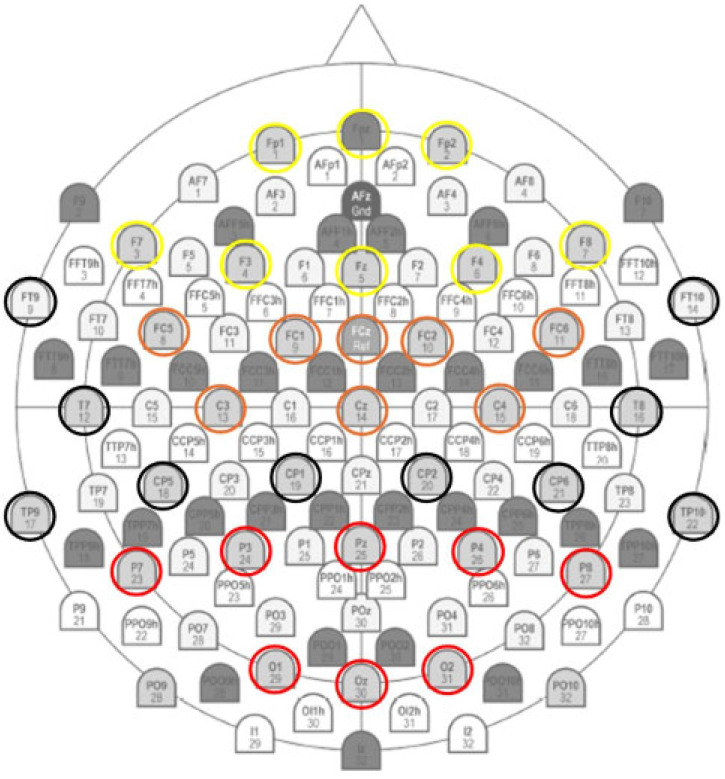
EEG setup. 32-channels electroencephalographic (EEG) set-up in 4 Region Of Interest (ROI) EEG: frontal region in yellow color (F: Fp1, Fpz, F3, F7, Fz, F4, F8, Fp2); fronto-central region in orange color (FC: FC5, FCZ, FC1, C3, Cz, C4, FC6, FC2); temporo-central region in black color (TC: FT9, T7, TP9, CP5, CP1, TP10, CP6, CP2, T8, FT10); parieto-occipital region in red color (PO: Pz, P3, P7, O1, Oz, O2, P4, P8).

Additionally, four key neuroanatomical ROIs were considered: the frontal region (F: Fp1, Fz, F3, F7, F4, F8, Fp2), the fronto-central region (FC: FC5, FCZ, FC1, C3, Cz, C4, FC6, FC2), the temporo-central region (TC: FT9, T7, TP9, CP5, CP1, TP10, CP6, CP2, T8, FT10), and the parieto-occipital region (PO: Pz, P3, P7, O1, Oz, O2, P4, P8) (Figure 2).

### 2.4. Statistical Data Analysis

Statistical analyses were performed on baseline-normalized PSD values using repeated-measures multivariate analysis of variance (MANOVA). Action Complexity (2: basic, advanced), ROI (4: F, FC, TC, PO), and Task (4: I, H, A, J) were entered as within-subject factors, while PSD values in the five frequency bands served as multivariate dependent variables. Significant multivariate effects were followed by univariate analyses and Bonferroni-corrected pairwise comparisons to identify the specific frequency bands and ROIs contributing to these effects, with a statistical threshold of *p* < 0.05.

When significant effects were observed, follow-up univariate analyses and pairwise comparisons were conducted for individual frequency bands. Effect sizes were systematically reported using partial eta squared (*ηp*^2^) to quantify the magnitude of observed effects. To control for multiple comparisons in post hoc analyses, the Bonferroni correction was applied. Assumptions of sphericity were assessed using Mauchly’s test; when violations of sphericity were detected, Greenhouse–Geisser corrections were applied to adjust the degrees of freedom. Prior to statistical analyses, data distribution was evaluated by inspecting skewness and kurtosis values, which indicated an adequate approximation to normality. All analyses were performed using SPSS (Statistical Package for the Social Sciences) version 28 (IBM Corporation, Armonk, New York, NY, USA).

The MANOVA design was explicitly structured to test the study hypotheses in relation to EEG patterns. The main effect of Complexity directly addressed the hypothesis that neural activity would vary as a function of assembly demands. The main effect of Task tested the hypothesis that the four elementary action stages would exhibit distinct neural profiles. The *Complexity* × *Task* interaction was specifically examined to verify whether task demands modulate neural responses differently across action stages, rather than producing a uniform effect. Furthermore, interactions involving ROI (*Complexity* × *ROI* and *Task* × *ROI*) were used to test hypotheses concerning the spatial organization of cortical activity.

## 3. Results

### 3.1. Behavioral and Self-Report Results

Behavioral reaction times (RTs) reflected condition complexity, with faster completion times for the basic assembly condition (M = 106 s) and slower completion times for the advanced assembly condition (M = 303 s), confirming that participants required more time to complete the more complex GOPA.

NASA-TLX total scores indicated that the basic-complexity condition was perceived as relatively low in mental workload (M = 24.3, SD = 19.6), whereas the more complex assembly condition elicited moderate workload (M = 46.9, SD = 21.0).

### 3.2. Multivariate Tests Results

Multivariate analysis of variance (MANOVA) was conducted to examine the effects of Complexity, Tasks, and ROI, as well as their interactions, on the set of dependent neural measures.

A significant main effect of Complexity was observed, using Pillai’s Trace [(V) = 0.160, F(5, 1180) = 44.88, *p* < 0.001, *ηp*^2^ = 0.160], which revealed that stimulus complexity significantly modulated the multivariate pattern of neural responses, with a moderate effect size.

A significant main effect of Task also emerged [V = 0.469, F(15, 3546) = 43.88, *p* < 0.001, *ηp*^2^ = 0.156], suggesting that neural activity varied robustly across task conditions.

The main effect of ROI was highly significant [V = 0.709, F(15, 3546) = 73.22, *p* < 0.0001, *ηp*^2^ = 0.236], reflecting marked regional differentiation in neural responses. Notably, this effect exhibited the largest effect size among the examined factors.

Regarding interaction effects, a significant *Complexity* × *Task* interaction was found [V = 0.038, F(15, 3546) = 3.04, *p* < 0.001, *ηp*^2^ = 0.013]. This finding indicates that the influence of stimulus complexity varies as a function of task demands.

A significant *Complexity* × *ROI* interaction was also observed [V = 0.255, F(15, 3546) = 21.98, *p* < 0.001, *ηp*^2^ = 0.085], suggesting that the effect of complexity was not spatially uniform but differed across cortical regions.

Similarly, the *Task* × *ROI* interaction reached statistical significance [V = 0.247, F(45, 5920) = 6.83, *p* < 0.001, *ηp*^2^ = 0.049], indicating region-specific modulation of neural activity depending on task condition.

In contrast, the three-way interaction (*Complexity* × *Task* × *ROI*) was not statistically significant [V = 0.029, F(45, 5920) = 0.778, *p* > 0.050, *ηp*^2^ = 0.006], suggesting the absence of a combined higher-order effect of the three factors on the multivariate neural response pattern. Overall, the results demonstrate that neural activity is significantly modulated by complexity, elementary tasks, and ROIs with selective interaction effects supporting region-specific and context-dependent functional modulation.

### 3.3. Univariate Tests Results

The following section reports the significant main and interaction effects for the univariate analysis (ANOVA).

#### 3.3.1. Delta Band (0.5–3.5 Hz)

First, a main effect for *Task* was observed [F(3, 1184) = 206.02, *p* < 0.001, *ηp*^2^ = 0.343]. Pairwise comparisons showed greater delta activity for the J compared to A, H, and I tasks (all *p* < 0.001). Also, higher delta power was observed for the A compared to the H and I tasks (*p* < 0.001).

Secondly, a main effect for *ROI* [F(3, 1184) = 77.45, *p* < 0.001, *ηp*^2^ = 0.164] was detected. As described in the pairwise comparisons, greater delta activity was found in the frontal compared to FC, TC, and PO (all *p* < 0.001). Also, greater values were found in FC compared to PO (p0 = 0.004), as well as TC compared to PO (*p* = 0.004).

**Figure 3:**
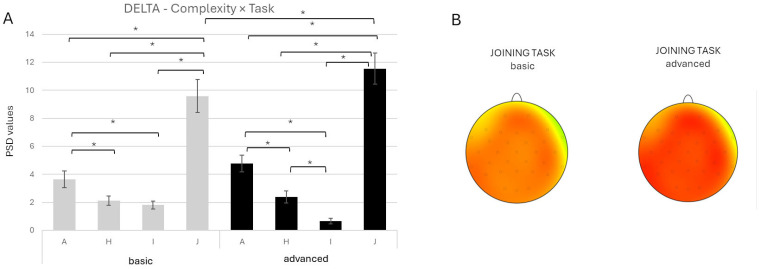
(**A**) Bar graph represents the significant *Complexity* × *Task* effect for the delta band. Bars represent ±1 standard error, and stars (*) mark statistically significant effects. (**B**) Topographical map of delta band (0.5–3.5 Hz) power during the basic complexity condition (left head) and the advanced complexity condition (right head) for the joining task. For topographic electroencephalographic (EEG) maps, the gray dots on the heads represent the location of the electrodes. The red represented an increase in power for the delta band during the joining task (software: Brain Vision Analyzer 2.0; GmbH, Gliching, Germany).

Thirdly, a significant *Complexity* × *Task* interaction effect was observed for the delta band [F(3, 1184) = 5.15, *p* = 0.002, *ηp*^2^ = 0.013]. As described in the pairwise comparisons, there was higher delta activity in the advanced compared to the basic-complexity condition during the J task (*p* < 0.001). Also, greater delta power was observed during the J task compared with the I, A, and H tasks across both complexity conditions (all *p* < 0.001). Additionally, the A task showed greater delta activity than I in the basic (*p* = 0.010) and advanced-complexity conditions (*p* < 0.001). Moreover, in both assembly conditions, the A task showed greater delta activity than the H task (all *p* < 0.001); only in the advanced-complexity condition, the H task show greater power than the I task (*p* = 0.018) (Figure 3A,B).

Finally, a significant *Task* × *ROI* interaction effect was also found [F(9, 1184) = 7.986, *p* < 0.001, *ηp*^2^ = 0.057], with post hoc comparisons confirming greater delta activity during the J task compared to the other elementary tasks across all ROIs (all *p* < 0.001).

#### 3.3.2. Theta Band (4–7.5 Hz)

For the theta band, a significant main effect for *Complexity* was observed [F(1, 1184) = 143.48, *p* < 0.001, *ηp*^2^ = 0.108], with greater theta power in the advanced compared to the basic-complexity condition (*p* < 0.001).

Then, a main effect for *Task* was identified [F(3, 1184) = 125.33, *p* < 0.001, *ηp*^2^ = 0.241], for which pairwise comparisons showed greater theta activity for the J compared to A, H, and I tasks (all *p* < 0.001). Also, greater values of the theta band were found for the A compared to the H and I tasks (*p* < 0.001).

Regarding the *ROI*, we found a significant main effect [F(3, 1184) = 229.94, *p* < 0.001, *ηp*^2^ = 0.368], with pairwise comparisons showing greater theta activity in the F ROI compared to FC, TC, and PO (all *p* < 0.001).

**Figure 4:**
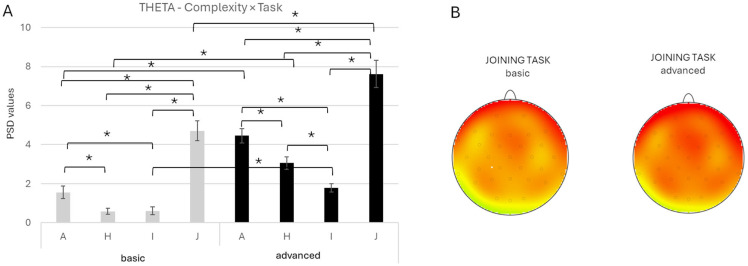
(**A**) Bar graph represents a significant *Complexity* × *Task* effect for the theta band. Bars represent ±1 standard error, and stars (*) mark statistically significant effects. (**B**) Topographical map of theta band power during the basic complexity condition (left head) and the advanced complexity condition (right head) for the joining task. For topographic electroencephalographic (EEG) maps, the gray dots on the heads represent the location of the electrodes. The red represented an increase in power for the theta band during the joining task (software: Brain Vision Analyzer 2.0, GmbH, Gliching, Germany).

Moreover, a significant *Complexity* × *Task* interaction emerged [F(3, 1184) = 4.309, *p* = 0.005, *ηp*^2^ = 0.011]. Comparisons showed higher theta activity in the advanced compared to basic-complexity conditions across all tasks (A: *p* < 0.001; H: *p* < 0.001; I: *p* = 0.003; J: *p* < 0.001). Also, greater theta values were found in the J task compared with the I, H, and A tasks across both complexity conditions (all *p* < 0.001). Additionally, the A task showed greater theta activity than H and I in both complexity conditions (all *p* < 0.001). In the advanced, the H task showed greater power than the I task (*p* = 0.018) (Figure 4A,B).

Interestingly, a significant *Complexity* × *ROI* interaction was observed [F(3, 1184) = 64.37, *p* < 0.001, *ηp*^2^ = 0.140]. Pairwise comparisons revealed higher theta activity in the F ROI compared with the FC ROI in both complexity conditions (*p* < 0.001). Moreover, the advanced compared to basic complexity condition elicited greater theta activity in the F (*p* < 0.001) and TC (*p* = 0.016) ROI.

Finally, a significant *Task* × *ROI* interaction effect was also found [F(9, 1184) = 2.45, *p* = 0.009, *ηp*^2^ = 0.018], with post hoc comparisons displaying greater theta activity during the J task compared to the other elementary tasks across all ROIs (all *p* < 0.001). Moreover, greater theta activity was observed in the F compared to FC, TC, and PO ROI across all tasks (all *p* < 0.001).

#### 3.3.3. Alpha Band (8–12.5 Hz)

About the alpha band, a significant main effect for *Complexity* was observed [F(1, 1184) = 31.65, *p* < 0.001, *ηp*^2^ = 0.026], with greater power in the advanced compared to the basic (*p* < 0.001).

Also, a main effect for *Task* was identified [F(3, 1184) = 222.55, *p* < 0.001, *ηp*^2^ = 0.361], for which pairwise comparisons showed greater alpha activity for the J compared to A, H, and I tasks (all *p* < 0.001). Also, greater values of the alpha band were found for the A compared to the H and I tasks (*p* < 0.001).

As far as the *ROI* is concerned, we found a significant main effect for the alpha band [F(3, 1184) = 23.70, *p* < 0.001, *ηp*^2^ = 0.057], with pairwise comparisons showing greater alpha activity in the F ROI compared to FC (*p* < 0.001), TC (*p* = 0.005), and PO (*p* < 0.001).

**Figure 5:**
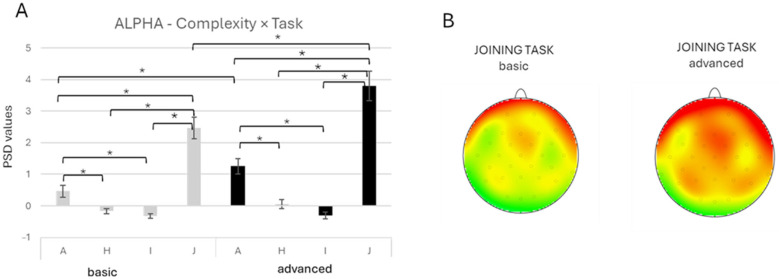
(**A**) Bar graph represents significant *Complexity* × *Task* effect for the alpha band. Bars represent ±1 standard error, and stars (*) mark statistically significant effects. (**B**) Topographical map of alpha band power during the basic complexity condition (left head) and the advanced complexity condition (right head) for the joining task. For topographic electroencephalographic (EEG) maps, the gray dots on the heads represent the location of the electrodes. The red represented an increase in power for the alpha band during the joining task (software: Brain Vision Analyzer 2.0, GmbH, Gliching, Germany).

A significant *Complexity* × *Task* interaction was found for the alpha band [F(3, 1184) = 7.76, *p* < 0.001, *ηp*^2^ = 0.019]. Pairwise comparisons indicated greater alpha activity during the J task compared with I, A, and H across both complexity conditions (all *p* < 0.001). The A task also showed higher activity than I (*p* = 0.016) and H (*p* = 0.001) in the basic, as well as in the advanced-complexity condition (all *p* < 0.001). Also, significantly greater alpha activity was found for the advanced compared to the basic-complexity condition during the A (*p* < 0.001) and J tasks (*p* < 0.001) (Figure 5A,B).

A significant *Task* × *ROI* interaction was also observed [F(9, 1184) = 3.75, *p* < 0.001, *ηp*^2^ = 0.028], reflecting higher alpha activity during the J compared to the other tasks across all ROIs (all *p* < 0.001).

#### 3.3.4. Beta Band (13–30 Hz)

For the beta band, a significant main effect for *Complexity* was observed [F(1, 1184) = 27.14; *p* < 0.001; *ηp*^2^ = 0.22], with greater power in the advanced-complexity condition compared to the basic-complexity condition (*p* < 0.001).

Then, a main effect for *Task* was identified [F(3, 1184) = 141.70; *p* < 0.001; *ηp*^2^ = 0.264], for which pairwise comparisons showed greater beta activity for the J compared to A, H, and I tasks (all *p* < 0.001). Also, greater values of theta band were found for the A compared to the H and I tasks (*p* < 0.001) and H compared with I (*p* < 0.001).

Regarding the *ROI*, we found a significant main effect [F(3, 1184) = 27.30; *p* < 0.001; *ηp*^2^ = 0.065], with pairwise comparisons showing greater beta activity in the TC ROI compared to F (*p* = 0.009) and FC (*p* = 0.004), and PO ROI with F and FC regions (*p* < 0.001).

**Figure 6:**
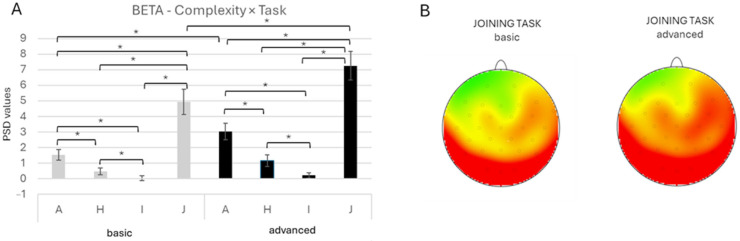
(**A**) Bar graph represents significant *Complexity* × *Task* effect for the beta band. Bars represent ±1 standard error, and stars (*) mark statistically significant effects. (**B**) Topographical map of beta band power during the basic complexity condition (left head) and the advanced complexity condition (right head) for the joining task. For topographic electroencephalographic (EEG) maps, the gray dots on the heads represent the location of the electrodes. The red represented an increase in power for the beta band during the joining task (software: Brain Vision Analyzer 2.0, GmbH, Gliching, Germany).

About the *Complexity* × *Task* interaction, it was significant [F(3, 1184) = 4.39, *p* = 0.004, *ηp*^2^ = 0.011]. Pairwise comparisons revealed greater beta activity during the joining task compared with the other tasks across both complexity conditions (all *p* < 0.001) and greater activity for J, as well as for A, compared between the basic and the advanced conditions (all *p* < 0.001). While the A task showed higher activity than Identification and H in both complexity conditions (all *p* < 0.001), with an increase in the difference in the advanced-complexity condition (*p* < 0.001) (Figure 6A,B).

A significant *Task* × *ROI* interaction was also found [F(9, 1184) = 4.26, *p* < 0.001, *ηp*^2^ = 0.031], with higher beta activity during the J task across all ROIs (all *p* < 0.001).

#### 3.3.5. Gamma Band (30.5–50 Hz)

For the gamma band, a significant main effect of *Complexity* was observed [F(1, 1184) = 20.38, *p* < 0.001, *ηp*^2^ = 0.017], with higher gamma power in the advanced-complexity condition compared to the basic-complexity condition.

A significant main effect of *Task* also emerged [F(3, 1184) = 171.80, *p* < 0.001, *ηp*^2^ = 0.303]. Pairwise comparisons indicated greater gamma activity during the J task compared to the A, H, and I tasks (all *p* < 0.001). In addition, the A task showed higher gamma power than both H and I (all *p* < 0.001), and H was greater than I (*p* < 0.001), reflecting a graded increase across task demands.

A significant main effect of *ROI* was found [F(3, 1184) = 138.71, *p* < 0.001, *ηp*^2^ = 0.260]. Post hoc comparisons revealed higher gamma activity in posterior regions, with greater power in the PO ROI compared to F, FC, and TC regions (all *p* < 0.001). Additionally, the TC ROI showed higher activity than the F and FC regions (all *p* < 0.001), indicating a posterior predominance of gamma-band responses.

**Figure 7:**
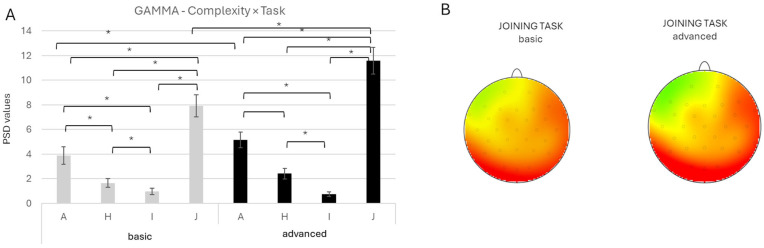
(**A**) Bar graph represents significant *Complexity* × *Task* effect for the gamma band. Bars represent ±1 standard error, and stars (*) mark statistically significant effects. (**B**) Topographical map of gamma band power during the basic complexity condition (left head) and the advanced complexity condition (right head) for the joining task. For topographic electroencephalographic (EEG) maps, the gray dots on the heads represent the location of the electrodes. The red represented an increase in power for the gamma band during the joining task (software: Brain Vision Analyzer 2.0, GmbH, Gliching, Germany).

The *Complexity* × *Task* interaction was significant [F(3, 1184) = 7.57, *p* < 0.001, *ηp*^2^ = 0.019]. Post hoc comparisons showed that gamma activity was higher in the advanced compared to the basic-complexity condition for the J task (*p* < 0.001) and, to a lesser extent, for the A task (*p* = 0.038). Across both complexity conditions, the J task consistently elicited higher gamma activity than all other tasks (all *p* < 0.001), while A remained higher than H and I (all *p* < 0.001) (Figure 7 A,B).

Finally, a significant *Task* × *ROI* interaction was observed [F(9, 1184) = 19.97, *p* < 0.001, *ηp*^2^ = 0.132]. Post hoc comparisons confirmed that the J task elicited higher gamma activity across all ROIs (all *p* < 0.001), with a spatial distribution emphasizing posterior regions.

## 4. Discussion

The present study examined the neurophysiological (EEG) bases of two ecologically valid GOPAs, investigating how complexity, task, and ROIs modulate EEG activity across frequency bands. Consistent with the literature on hierarchical actions [1,2,3], the results show that neural activity associated with manual assemblies emerges as an integrated and distributed pattern rather than as the sum of local or band-specific effects.

Multivariate results indicate that complexity, task, and ROI modulate the multivariate pattern of responses, reflecting both complexity and task-specific effects and regional heterogeneity. The main effect of complexity suggests that increasing task demands systematically alter EEG-dependent measures (theta, alpha, beta, and gamma bands), consistent with models of cognitive load–dependent modulation. Behavioral and self-report results align with condition complexity, suggesting that increased component number, sequencing demands, and coordination requirements in the advanced complexity condition led to higher cognitive and attentional engagement. Elementary task effects indicate distinct multivariate profiles across different conditions (with a key role for J and A tasks). While the ROI effect underscores functional specialization of the examined regions, with anterior regions more involved for low-frequency bands and posterior ROIs engaged for high-frequency bands. Overall, these findings highlight both the global effects of complexity on EEG frequency bands and brain region-specific contributions based on complexity and elementary tasks.

The first key finding concerns the fact that increased complexity does not uniformly affect all tasks but selectively amplifies differences among them. Specifically, for J and A tasks, there was an increase across all EEG frequency bands for the advanced compared to basic-complexity conditions, suggesting that certain elementary tasks are more sensitive to increased cognitive load than others. This result is consistent with action control models distinguishing between more automatic tasks and tasks requiring stronger executive system involvement [11]. In particular, the J task consistently exhibited higher activity levels than other tasks (I, H, A), regardless of complexity, suggesting that it requires greater integration of planning, control, and motor implementation. Moreover, the A task exhibited higher activity levels than I and H, with differences among complexities. These findings align with hierarchical conceptions of action [7,8], according to which complex actions are executed as organized sequences of sub-actions. The analyzed assembly conditions, structured into I, H, A, and J phases, appear to differ not only in physical demands but also in the relative weight of cognitive and decisional processes and reactions involved at each stage.

Different elementary assembly tasks showed distinct oscillatory patterns consistent with their functional roles. The J task elicited the strongest and most widespread activation across frequency bands and regions, indicating that it represents the most cognitively and sensorimotor-demanding subtask. J likely requires simultaneous integration of spatial alignment, force control, and goal monitoring, thereby engaging both executive and motor networks. A showed intermediate activation, whereas I and H were associated with comparatively lower-frequency activity. This graded neural pattern mirrors the increasing integrative and coordinative demands of the elementary tasks, supporting hierarchical models in which complex actions are decomposed into functionally differentiated components. More specifically, the stronger EEG association observed for A and, even more prominently, for J may reflect the need to coordinate multiple sensorimotor and cognitive processes in parallel. A requires continuous spatial adjustment and planning, engaging visuospatial mechanisms, whereas J further adds action control sequencing and verification of assembly stability. In contrast, I and H involve more isolated operations with lower integrative demands. Consequently, A and especially J impose greater neural integration, which may explain their stronger EEG signatures, particularly in the more complex assembly task where component interdependencies increase cognitive-motor load.

Importantly, the present results reveal a dissociation between motor demands and neural activity. Despite being one of the most motorically active phases, H elicited lower oscillatory power compared to J and A across multiple frequency bands. This pattern indicates that neural activity does not scale linearly with the physical demands of the task. Instead, higher oscillatory power appears to reflect increased executive engagement, including action planning, monitoring, and sensorimotor integration. H may rely more heavily on relatively automatized motor routines, whereas J and A require continuous updating of action goals and motor performance monitoring. This dissociation supports the interpretation that EEG oscillatory dynamics primarily index the cognitive and integrative demands of action execution rather than mere motor output, reinforcing the distinction between physical and neurocognitive workload in complex goal-directed behavior.

Notably, complexity selectively amplified neural activity in the more demanding tasks, rather than uniformly affecting all stages. This selective increase aligns with models of visual control, suggesting that increasing assembly complexity disproportionately affects stages that require higher-order integration and monitoring. In applied contexts, this implies that neural markers sensitive to workload may be particularly informative when targeting critical assembly stages, rather than the entire task block. Consequently, assembly complexity may reveal latent differences in the cognitive structure of assembly operations that remain less evident under simpler conditions.

Furthermore, the second key result concerns the role of the EEG theta band in the context of this study. Our findings indicate that assembly complexity specifically modulates this frequency band within the target ROIs. A significant effect emerged for the theta band, whereas no comparable effects were observed in the higher EEG frequency bands. Specifically, greater theta power was observed in the frontal ROI compared to the fronto-central ROI during both complexity conditions. Moreover, when comparing the advanced-complexity condition to the basic one, increased theta activity was specifically detected in frontal and temporo-central regions. These findings support the interpretation of the theta band as a marker of cognitive control during complex conditions. In particular, frontal theta appears to represent a sensitive index of mental workload and adaptive control in goal-directed, tool-mediated actions. The observed increase in theta activity is consistent with the well-established role of this band in cognitive control, performance monitoring, and the allocation of attentional resources during tasks that require continuous action supervision [20]. Frontal theta activity has been widely associated with performance monitoring and attentional allocation [23], and the present results extend this interpretation to ecologically valid, tool-mediated manual tasks.

Thirdly, across all elementary tasks, we observed a specific topographical configuration varying by frequency band. EEG activity was consistent with the anterior–posterior functional gradient discussed in the introduction [14]. Frontal regions showed greater activity in low-frequency bands (delta, theta, and alpha), whereas temporo-parietal and occipital regions were more involved in high-frequency bands (beta and gamma). This distribution supports the interpretation that anterior areas are primarily engaged in planning, monitoring, and cognitive control, while posterior regions support sensorimotor integration, spatial processing, and object representation necessary for accurate manipulation [1,3].

Moreover, the J task again showed widespread involvement across all ROIs, whereas other tasks exhibited more selective patterns with differential anterior–posterior distribution.

However, alternative explanations should be considered. Differences in oscillatory activity may partly reflect variability in movement execution, strategy use, or residual motor-related artifacts, particularly in ecological tasks involving continuous manual actions. Although the observed dissociation between motor demands and EEG activity supports a cognitive interpretation, the absence of direct control measures (e.g., EMG or motion tracking) limits the ability to fully disentangle cognitive and motor contributions. Moreover, the susceptibility of high-frequency bands to muscular contamination further constrains the interpretation of beta and gamma effects. These methodological aspects should be considered when evaluating the strength and specificity of the present findings.

Overall, these findings support the interpretation that EEG oscillatory dynamics provide sensitive markers of both assembly complexity conditions and elementary task-specific demands. Rather than reflecting generalized arousal, neural modulation tracked the hierarchical and functional structure of goal-directed actions. From an applied neuroscience perspective, these results support the integration of frequency-specific EEG measures into neuroergonomic assessments of real manual actions. Such markers may inform the design of adaptive systems, workload monitoring protocols, and training strategies aimed at optimizing performance and reducing cognitive overload in human-centered manufacturing environments.

Importantly, these findings extend beyond a mere description of task-related differences by suggesting that EEG oscillatory activity reflects the dynamic organization of cognitive control processes underlying goal-directed actions. Rather than indexing a unitary increase in workload, the observed neural patterns indicate that different elementary tasks recruit partially distinct functional configurations depending on their specific computational demands. This supports the view that cognitive control operates as a flexible and context-dependent system, in which neural resources are dynamically reconfigured to support planning, monitoring, and sensorimotor integration processes. In this sense, the present results provide empirical support for models of hierarchical action control, extending them to ecologically valid, tool-mediated tasks.

A relevant direction for future research is to move beyond the current coarse-grained task decomposition and adopt a more fine-grained analytical approach. Specifically, each task stage could be further decomposed into elementary processing or movement units, allowing for the identification of distinct neural signatures associated with specific sub-components. This approach is supported by previous EEG decoding studies showing that neural activity can reliably encode detailed movement parameters such as direction, distance, and spatial position [28]. Extending this perspective to the present paradigm would enable a more precise interpretation of the observed effects, clarifying which specific cognitive or motor processes drive the differences in brain signals. Moreover, integrating methodologies from the EEG decoding and brain–computer interface literature, where increasingly fine-grained features are extracted from neural signals, could further enhance the sensitivity and interpretability of future analyses.

Beyond statistical significance, the observed oscillatory modulations demonstrate meaningful functional relevance for real-world applications. The magnitude and consistency of the effects, particularly in frontal theta and posterior beta–gamma activity, suggest that EEG markers can reliably track variations in cognitive workload and sensorimotor integration during complex manual tasks. From a neuroergonomic perspective, these findings support the potential use of EEG-based metrics for real-time monitoring of operator workload, identification of cognitively demanding task phases, and the development of adaptive systems aimed at optimizing performance and reducing overload. Such applications are particularly relevant in human-centered manufacturing and training environments, where understanding the neurocognitive demands of specific task components may inform more efficient task design and support strategies.

## 5. Conclusions

The present findings demonstrate that EEG oscillatory dynamics during ecological GOPAs are selectively modulated by both action complexity and the functional organization of elementary tasks.

Increased complexity did not produce a uniform enhancement of neural activity; rather, it selectively amplified oscillatory power during the J and, most prominently, A tasks, which consistently elicited higher activity across frequency bands and ROIs. This pattern indicates that J represents the stage at which executive monitoring, spatial evaluation, and fine motor implementation converge most strongly within hierarchical action sequences.

Complexity-related effects were primarily expressed in the theta band, with enhanced frontal and temporo-central theta power in the advanced-complexity condition, reinforcing the role of frontal theta as a sensitive index of adaptive cognitive control and mental workload regulation during extended, tool-mediated manual actions [22]. The observed anterior–posterior gradient further supports a differentiated cortical contribution, with frontal regions preferentially involved in lower-frequency activity associated with executive supervision [14] and posterior regions supporting higher-frequency dynamics related to sensorimotor integration [3,21].

Overall, these results substantiate a neuroergonomic account of praxis, in which behavior emerges from the dynamic interaction between cognitive control, sensorimotor integration, and task structure [12,13]. By integrating EEG frequency bands, cortical ROIs, complexity manipulation, and elementary task decomposition, the study provides a fine-grained neurophysiological mapping of GOPAs and addresses the gap in the literature concerning multilevel and ecologically valid investigations of hierarchical action.

Despite the novelty of this work, the following limitations should be acknowledged. First, only two assembly tasks within a similar mechanical domain were employed, restricting generalizability to other goal-oriented actions, such as conditions with tool use, requiring bimanual coordination, or non-mechanical assemblies.

Although the sample size was adequately powered for the planned repeated-measures MANOVA, the study involved a relatively homogeneous group of healthy young adults, which may limit generalizability to broader populations, including older individuals or clinical groups characterized by praxis impairments. Moreover, future studies should further investigate cognitive flexibility and how individual differences in motor experience, lateralization, and executive functioning may modulate EEG patterns during complex action tasks. Second, the use of a 32-channel EEG system, while appropriate for ecological and applied settings, constrains the spatial resolution of source localization and limits precise inferences regarding underlying cortical generators, with a lack of analysis of within-task temporal dynamics. Due to the variability in the timing and frequency of elementary actions across trials and participants, it was not possible to reliably align and segment task phases (e.g., planning vs. execution). Consequently, the reported results reflect averaged activity over entire task segments, potentially overlooking finer-grained temporal fluctuations in cognitive and sensorimotor processes. Future studies integrating higher-density EEG, multimodal neuroimaging, behavioral performance metrics, and individual-difference measures of EFs may further refine the neurocognitive characterization of complex GOPAs. Although GOPAs were randomized in order, no analysis on potential order effects was provided. Given the differences between conditions, duration, fatigue, and practice-related carry-over effects may represent plausible confounds that should be formally tested in future studies. Future work could benefit from the integration of automated approaches (e.g., motion tracking, wearable sensors, or machine-learning-based action segmentation) to enhance temporal accuracy and reproducibility. Also, additional artifact correction (e.g., ICA with movement regressors, EMG monitoring) or concurrent motion capture would help disentangle genuine neural oscillations from movement-induced noise in future studies. Moreover, the present design does not allow causal inferences regarding the role of EEG oscillations, which should be addressed in future studies using perturbation approaches or computational modeling.

From an applied perspective, these findings suggest that assembly complexity should be evaluated not only in terms of physical workload but also in relation to neurocognitive processes that support action execution, as well as its elementary tasks. This evidence may inform the human-centered design of tools, training protocols, and collaborative robotic systems aimed at supporting phases associated with higher executive workload. Furthermore, we speculate that in clinical contexts, praxis frameworks based on manual action decomposition and oscillatory dynamics could guide refined assessment and intervention strategies also for clinical conditions such as ADHD [29], autism spectrum disorder [30], and Tourette syndrome [31]. Indeed, a promising speculative direction involves integrating EEG-derived markers into adaptive systems capable of monitoring cognitive workload in real time, dynamically modulating task demands, or providing tailored support to the operator. Future studies combining high-density EEG, multimodal imaging, behavioral metrics, and individual-difference indices of executive functions will further clarify the neurocognitive architecture of complex goal-directed actions.

## Data Availability

The data presented in this study are available on request from the corresponding author due to ethical reasons for sensitive personal data protection (requests will be evaluated according to the GDPR—Reg. UE 2016/679 and its ethical guidelines).

## Abbreviations

The following abbreviations are used in this manuscript:

GOPA	Goal-oriented praxis actions
EF	Executive-cognitive functions
ROI	Region of Interest
F	Frontal
FC	Fronto-central
TC	Temporo-central
PO	Parieto-occipital
EEG	Electroencephalography
PSD	Power spectral density
I	Identification
H	Handling
A	Alignment
J	Joining
